# Arabic translation, cross-cultural adaptation, and validation of the Expectation for Treatment Scale (ETS) in patients with musculoskeletal disorders

**DOI:** 10.1371/journal.pone.0346025

**Published:** 2026-03-27

**Authors:** Walid Mohamed, Michelle Hall, Jürgen Barth, Paul Hendrick

**Affiliations:** 1 School of Health Sciences, University of Nottingham, Nottingham, United Kingdom; 2 School of Health Sciences, Queen’s Medical Centre, Nottingham, United Kingdom; 3 Complementary and Integrative Digital Health, Institute of Primary Care, University of Zurich and University Hospital Zurich, Zurich, Switzerland; University of Hafr Al-Batin, SAUDI ARABIA

## Abstract

**Purpose:**

To translate, culturally adapt the Expectation for Treatment Scale (ETS) into Arabic and evaluate its psychometric properties in patients with musculoskeletal disorders.

**Methods:**

Following established guidelines, forward and backward translations were performed, each followed by a synthesis. Face validity of the Arabic ETS was evaluated by asking patients undergoing physiotherapy for musculoskeletal disorders to what extent the ETS items covered their outcome expectations. Internal consistency; test-retest reliability; measurement error; content validity; construct and structural validity, and floor and ceiling effects were assessed.

**Results:**

The ETS was successfully translated and adapted according to the 24 patients’ feedback. 205 individuals completed the online questionnaire, and 36 completed it twice. The Arabic ETS demonstrated good internal consistency (α = 0.75), high test-retest reliability (ICC = 0.93 and κw = 0.75, 0.58, 0.55, 0.66, 0.70, for items one to five, respectively), low measurement error (SEM = 1.42, and SDC = 2.86), and acceptable content validity (0.78). Construct validity was supported (though not definitive) via known-group hypothesis testing (r = −0.331, p < .001) and exploratory factor analysis (χ²(66 = 218.73, p < 0.001). Differential item functioning confirmed the cross-cultural validity. There were minimal floor (0.5%) and ceiling (2%) effects.

**Conclusions:**

The ETS was translated and adapted to Arabic culture. The assessed psychometric properties of the Arabic ETS support its reliable use in patients with musculoskeletal conditions.

## Introduction

Musculoskeletal disorders (MSDs) impose a significant burden globally [1]. MSDs are linked to physical, psychological, and organisational dysfunction, leading to reduced productivity and high healthcare costs, so imposing substantial well-being and financial burdens on both individuals and societies [2,3]. MSDs impact more than 25% of the world population, representing 21% of global morbidity [4] and are a leading cause of disability [1]. Accounting for 5.6% of total disability-adjusted life years and 15.9% of total disability years, MSDs are the fifth most common cause of disability-adjusted life years and the primary cause of years lived with a disability [5,6].

MSDs are associated with physical, psychological, and organisational dysfunction, resulting in decreased productivity and significant healthcare costs, imposing significant wellness and financial burdens on individuals as well as societies [2,3]. Psychosocial factors, such as patient expectations, have been shown to affect the progression and worsening of MSDs [7,8]. Patient expectations are individuals’ beliefs concerning the likelihood of future events [9]. Patient expectations can be classified into two primary categories: outcomes and treatment expectations [10,11]. Outcome expectations refer to an individual’s beliefs for advancement, and treatment expectations relate to their anticipatory beliefs regarding the nature of the experience, including expectations around the therapeutic alliance [12,13].

Previous research has identified an association between outcome expectations and treatment outcomes in individuals with musculoskeletal pain conditions [14,15]. A recent systematic review reported strong evidence that positive recovery expectations are associated with improved return-to-work outcomes in individuals with musculoskeletal pain conditions [15] and that patients with low (negative) outcome expectations were twice as likely to have work disability than those with higher (or more positive) expectations (OR = 2.06 [95% CI 1.20–2.92] P, 0.001) [15]. However, issues with measuring expectations are a substantial limitation in the research [16,17].

Heterogeneity in ways of measuring patient expectations was found to be a restraint in many systematic reviews and meta-analyses exploring outcome expectations [14,18–20]. This entails, for example, variations in the conceptualisation and definition of patients’ expectations [12] and the heterogeneity or the lack of a theoretical framework for expectation measures [19]. This heterogeneity, as well as the lack of a theoretical structural framework, may impact the quality of expectations measures [17]. Additionally, psychometric properties of outcome expectations measures are not well-investigated [21], with the majority of these measures lacking both conceptual consistency and proper psychometric assessment [17].

Only a limited number of studies have examined patient expectations within Arabic culture [22–25]. Mostafa [22] examined patient expectations concerning the quality of services provided by 12 hospitals. Al Fraihi et al. [23] investigated patient expectations regarding outpatient care in hospitals. Hasan et al. [24] examined anticipations regarding primary care pharmacies in the United Arab Emirates. Gaarslev et al. [25] assessed expectations for participants with upper respiratory tract infections. Collectively, these studies predominantly focused on expectations about service quality and certain aspects of healthcare delivery, rather than on patients’ expected outcomes.

None of these studies [22–25] investigated outcome expectations or employed a psychometrically validated Arabic instrument for measuring such expectations. The scarcity of research examining outcome expectations for Arabic-speaking populations may result from the absence of psychometrically validated instruments. Therefore, it is crucial to develop a measure of outcome expectations for Arabic-speaking cultures.

According to Krogsgaard et al. [26], it is advisable to translate and adapt an existing measure rather than developing a new one if there are already psychometrically sound measures that were developed to measure the construct of interest for the same population [26]. Additionally, cross-cultural considerations must be taken into account when translating self-reported measures for use in a distinct culture [27]. Considering the absence of a psychometrically validated measure to assess outcome expectancies for Arabic speakers and that reliable measures have been developed in other languages, translating and culturally adapting an existing measure is a logical strategy.

The Expectation for Treatment Scale (ETS) is a newly developed generic measure that measures patients’ outcome expectations [21]. The ETS is a short, easy-to-administer measure that explicitly focuses on patients’ outcome expectations. It was initially developed in German and translated into English by its developers. The ETS has been previously validated in clinical populations, and it has been utilised in different settings [28–30], which arguably supports its suitability for adaptation into new cultural contexts. Therefore, this research aims to translate and cross-culturally adapt the ETS [21] for use in Arabic culture, and evaluate its psychometric properties in patients with musculoskeletal disorders.

## Methods

### Study design

**Fig 1:**
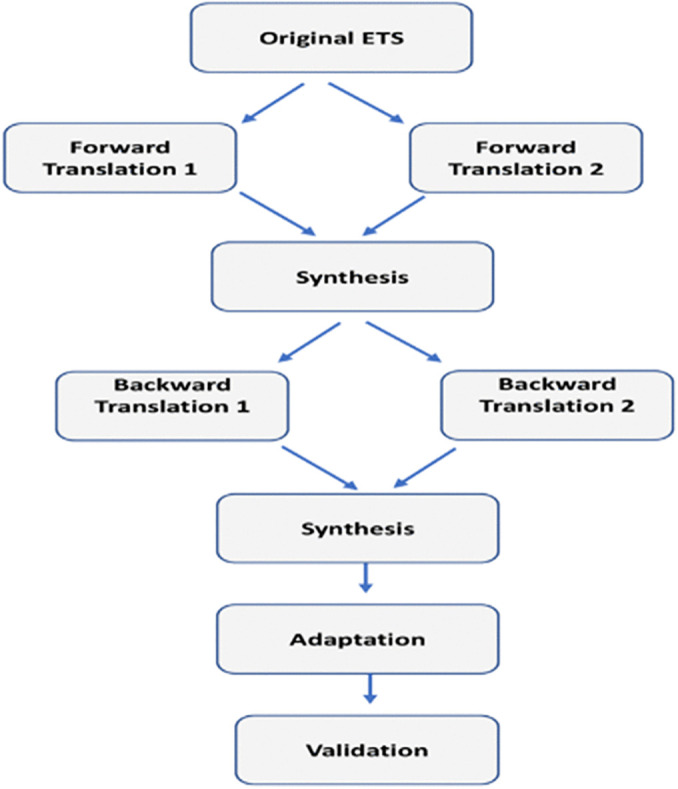
Steps for the translation of the ETS, adapted from Tsang et al. [23].

Following a set of steps, this study employed Tsang et al.'s [27] guidelines for the translation, cultural adaptation, and validation of measures. These steps included, forming an expert committee, forward translation, synthesis (committee meeting), back translation, synthesis (committee meeting), pilot testing (interviews for adaptation), adapting the Arabic ETS and psychometric evaluation (Fig 1).

Face validity of the translated ETS was evaluated qualitatively during the adaptation phase [27]. Additionally, participants were requested to fill in the questionnaire to assess its psychometric properties. Participants who completed the translated ETS were asked if they would like to fill it in again after two weeks for the test-retest reliability assessment [31].

Interviews were conducted between 08/11/2023 and 10/01/2024. Whereas data collection for the validation phase took place between 21/03/2024 and 29/03/2024 for physiotherapist and between 21/03/2024 and 20/06/2024 for patients with retest reliability evaluated until 27/06/2024.

### Ethical considerations

Permission was obtained prior to the study from the developer of the original scale. The Research Ethics Committee at the School of Health Sciences, University of Nottingham, reviewed this study and issued a favourable opinion (Ref: FMHS 322-0723). Informed consent for the study was obtained in two ways: written consent for the interviews, and electronic consent at the beginning of the online questionnaires for both patients and physiotherapists.

### Participants and recruitment

This study involved contacting the heads of physiotherapy departments at three hospitals in Libya to help facilitate access and recruit potential participants and act as clinical gatekeepers. Patients were recruited from: Althahara Centre for Rehabilitation, Althahara, Bani Waleed, Libya; Alzawya Centre for Physical Therapy, Al Tariq Al Sahili, Joud Dayim, Alzawya, Libya; Medical Centre for Physiotherapy, Misrata, Libya. To be included, patients must be adults (>18) receiving physiotherapy for musculoskeletal disorders, be literate native Arabic speakers, and be able to read Arabic and be willing to give informed consent.

**Fig 2:**
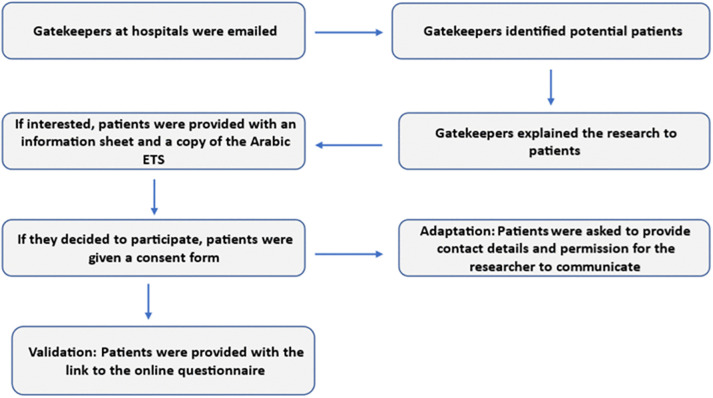
Flow diagram showing the study processes.

The gatekeeper informed potential participants about the research study, and those interested were given a detailed study information sheet. Informed consent was obtained by the clinical gatekeepers and patients received a copy of the Arabic ETS and a link to an online questionnaire. The clinical gatekeeper obtained the patient’s permission for the research team to schedule interviews. Fig 2 provides a flow diagram of patients’ recruitment process.

For content validity evaluation, physiotherapists at the three hospitals were also invited to take part in a content validity assessment of the Arabic ETS by completing an online questionnaire. This questionnaire included: an overview of the research, approval of receiving the information sheet and the consent form, the Arabic ETS, and a Likert scale (from 1 to 4) beneath each item to rate its comprehensiveness and coverage of outcome expectations.

The sample size for the adaptation phase (interviews) and for the validation was determined by the guidelines developed by Tsang et al. [23], for the translation, adaptation and validation of self-reported measures. The advised sample sizes for culturally adapting a translated measure are generally between 30–50 individuals [27], whereas for the validation process, a sample size of 200 is deemed an acceptable threshold, and 300 individuals is deemed sufficient to produce more reliable and generalisable findings [27].

### Translation

The translation committee included the following members: the four authors, including a subject matter expert (Dr JB, the corresponding author of the original questionnaire), two forward translators, two backward translators, and participants representing Patient and Public Involvement (PPI). A bilingual patient with a musculoskeletal disorder and a bilingual member of the public in Libya.

One forward translator received a document containing information about the conceptual foundation for the items [32,33], which was revised and approved by the original questionnaire developer. It provides descriptions and explanations of the key terms and constructs used in questionnaire items. McKown et al. [34] state that the concept definition document contains information regarding the conceptual basis of each item or task. All four translators worked independently and were asked to submit their translations in a document together with any relevant comments or notes concerning the translation process.

Aiming at resolving inconsistencies between the translations, a synthesis followed each translation. The translators’ reports were reviewed multiple times in advance to identify specific points of disagreement that could be addressed during the syntheses. All decisions, agreements, and modifications made during the syntheses were documented to ensure transparency and serve as a resource. Following backward translation synthesis, the committee was provided with a written report addressing each concern, and recognising the steps taken.

### Cross-cultural adaptation

**Fig 3:**
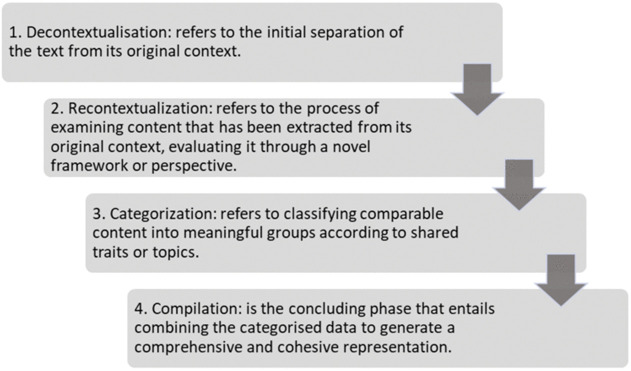
Four stages for arranging and completing qualitative analyses employing content analysis by Bengtsson [33].

The data included non-verbatim Arabic transcriptions of the semi-structured interviews. The interview consisted of a set of questions, with additional prompt questions (S1 Appendix). Directed content analysis was used to identify data patterns using a pre-established set of codes [35]. Directed content analysis is flexible and allows for code adjustments and additions based on analysis, improving research transparency [36]. Bengtsson [37] outlines a framework for qualitative content analysis comprising four essential steps: Decontextualisation, Recontextualization, Categorisation, and Compilation (Fig 3).

Initially, each Arabic transcription was reviewed and sentences deemed pertinent to the interviews’ aims and objectives were extracted as quotes into a table, accompanied by initial themes. Analysis of these tables was conducted to synthesise and understand the themes and patterns. Subcategories were formulated via analysing the data within each category based on corresponding characteristics or themes. Thereafter, these tables were translated by a bi-lingual researcher and subsequently shared anonymously with a professional translator for verification of translation accuracy.

The proposed modifications were discussed by the adaptation committee to the questionnaire based on participant feedback [38,39]. The adaptation committee consisted only of the four authors. Two criteria must be met to reject a proposed modification during the cross-cultural adaptation process: A. The adaptation committee must deem the change irrelevant and insignificant concerning outcome expectations; and B. Less than 20% of the participants indicated the change [40].

### Validation

The face validity of the Arabic ETS was evaluated by asking participants to what extent they thought the questionnaire items covered their outcome expectations [27]. According to Streiner et al. [41, p. 80], face validity pertains to the views of the scale’s respondents and it is therefore evaluated by those respondents, rather than by subject matter experts.

**Fig 4:**
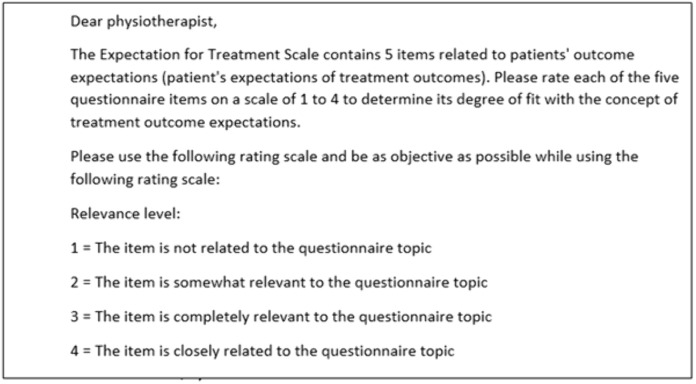
Rating instructions for content validity assessment.

Content validity was evaluated by calculating scale and item level content validity indexes (S-CVI and I-CVI). Physiotherapists were asked to rank each item on 1–4, as suggested by Yusoff [42], the content validity form (Fig 4) was shared with the experts (physiotherapists) to ensure that the experts understood the task. To assess the construct validity of the Arabic ETS scale, three methodologies were employed: hypothesis testing for known-group validity (younger individuals would possess higher expectations), exploratory factor analysis (EFA) for structural validity and Chi-Square test for Differential Item Functioning (DIF) to evaluate cross-cultural validity.

For known-groups validity, a bivariate correlation analysis was conducted to investigate the correlation between age and the level of outcome expectation. It was hypothesised that younger patients typically have higher outcome expectations [43]. KMO of 0.87 and Bartlett’s test p < 0.001 confirmed sampling adequacy for EFA. Principal axis factoring extraction method was performed using direct Oblimin rotation. Factors were retained based on eigenvalues > 1 and inspection of the scree plot. For DIF, the Chi-Square test of independence was employed, analysing response distributions across groups (gender) by comparing responses between males and females.

Reliability has been evaluated in this research using three approaches. The internal consistency was assessed using Cronbach’s Alpha. The test-retest reliability was assessed in two ways; Intraclass Correlation Coefficient (two-way random-effects model with absolute agreement) [44] for total scores and Cohen’s Weighted Kappa for the items. In response to interviewees’ comments indicating perceived similarity, some items (1, 2 and 5), inter-item correlation coefficients (Pearson’s r) were computed to evaluate the extent of overlap between items.

Lastly, agreement property (measurement error) was evaluated by calculating the standard error of measurement (SEM _agreement_) and the smallest detectable change (SDC) [45]. The SEM was calculated manually using the formula SEM = SD × √(1 − α) [45], and the SDC was calculated using the formula SDC = 1.96 × √2 × SEM [45]. Floor and ceiling effects were evaluated by calculating the frequencies of the minimum and maximum possible scores (20 and 5) using SPSS.

## Results

### Translation results

Both forward translators provided their reports. During the first synthesis, for each wording difference, a term was chosen from the two alternatives already presented in the forward translation reports, therefore, no changes were made to the reports in terms of adding any new alternative words or terms. This has led to a consensus and ensured cultural compatibility with the Arabic-speaking demographic (S2 Appendix). Similarly, backward translators provided their reports. During the translation committee meeting, the original developer of the questionnaire expressed no objections to the alternatives regarding the terminology proposed by the backward translators. One difference between the two backward translation reports was the utilisation of present versus future tenses. This was discussed and the Arabic-speaking members contended that the present tense was also acceptable; however, the developer had a preference towards the future tense to clearly indicate that the items pertained to the future, as it was asking about expectation. Therefore, the decision was made to employ the future tense for all items.

There was some disagreement in the response options, with one translator employing “to some extent” and the other using “somewhat”, “to some extent disagree” versus “somewhat disagree”, for example. The translation committee agreed that either option effectively conveys the intended meaning of the original response options (partially), agreeing on the phrase “to some extent.” However, the questionnaire’s developer recommended using only “Disagree” instead of “to some extent disagree.” The rationale behind this recommendation was that “to some extent disagree” and “to some extent agree” were both viewed as overlapping, with both falling in between total agreement and total disagreement. Consequently, the committee resolved to substitute “to some extent disagree” with “Disagree” in the Arabic version of the questionnaire.

### Interview analysis results

The 24 interviewees included 10 females (41.7%) and 14 males (58.3%). The interviews were conducted in Arabic using Microsoft Teams and audio recorded. Table 1 outlines the characteristics of the participants and the details of the interviews. All participants consistently expressed that the overall questionnaire language was straightforward, except for Participant 8. The terminology utilised in the Arabic ETS was understandable to all participants except for Participant 10, who stated that item 5 was not sufficiently explicit. Five out of 24 participants (20.8%) unanimously suggested replacing the term أفضل (Better) with أقل (Less) for item five. They contended that the phrase “شكواي ستكون أفضل بكثير” (my complaint will be considerably better) would be interpreted as “having more complaints.”

**Table 1:** Characteristics of the participants and the specifics of the interviews.

Participant	Age	Sex	Location	Disorder	Education	Stage of treatment	Duration	Date
1	35–39	M	Misrata	Low Back Pain	Secondary School	At the start	08:46	11/11/23
2	40–44	M	Misrata	Cervical Spondylosis	BSc	Started	08:16	12/11/23
3	40–44	M	Bani Waleed	Carpel Tunnel Syndrome	Diploma	Started	07:47	12/11/23
4	40–44	M	Bani Waleed	Patellar Fracture	BSc	At the start	07:16	13/11/23
5	45–49	M	Misrata	Finger Fracture	BSc	At the start	06:34	14/11/23
6	60–64	M	Bani Waleed	Finger Fracture	High School	Started	18:44	17/11/23
7	30–34	M	Bani Waleed	Lateral and Medial Meniscus Tear	BSc	Started	10:22	18/11/23
8	55–59	M	Misrata	C3-4 and C4-5 Disc Herniation	PhD	Started	22:29	20/11/23
9	25–29	F	Misrata	Elbow Fracture	High School	At the start	09:26	29/11/23
10	30–34	F	Misrata	Knee Osteoarthritis	MSc	At the start	13:21	01/12/23
11	65–69	F	Misrata	Low Back Pain	Secondary school	At the start	09:01	03/12/23
12	35–39	M	Misrata	L4-5 Disc Prolapse	BSc	At the start	09:41	03/12/23
13	18–24	F	Misrata	Ankle Sprain	High School	At the start	07:11	06/12/23
14	18–24	F	Misrata	Wrist Sprain	High School	At the start	06:20	07/12/23
15	35–39	M	Bani Waleed	Anterior Cruciate Ligament	BSc	At the start	09:45	09/12/23
16	50–54	M	Bani Waleed	Duchenne Muscular Dystrophy	BSc	At the start	11:27	15/12/23
17	70–74	M	Misrata	Total Knee Replacement	High School	At the start	04:48	16/12/23
18	45–49	F	Misrata	Tennis Elbow	BSc	At the start	05:04	17/12/23
19	18–24	F	Bani Waleed	Multiple Metatarsal Fractures	BSc	At the start	10:03	17/12/23
20	35–39	M	Misrata	Tibial Plateau Fracture	BSc	At the start	06:42	17/12/23
21	35–39	F	Misrata	Low Back Pain & Knee Osteoarthritis	BSc	At the start	05:31	18/12/23
22	25–29	M	Bani Waleed	Low Back Pain	BSc	At the start	06:01	23/12/23
23	18–24	F	Bani Waleed	Postural Kyphosis	High School	At the start	06:03	01/01/24
24	30–34	F	Bani Waleed	Low Back Pain	BSc	At the start	07:15	10/01/24

Note: age ranges were used to avoid the possibility of identifying participants.

All participants reported finding it easy to select response options and respond to inquiries. Only Participant 8 commented on response options and proposed the inclusion of an “I don’t know” option despite acknowledging that the current options are suitable.

Only two participants (8.35%) offered additional feedback regarding the Arabic ETS. Participants 8 and 11 suggested the inclusion of a phrase that specifies that this questionnaire is administered before treatment.

### Adaptation results

**Fig 5:**
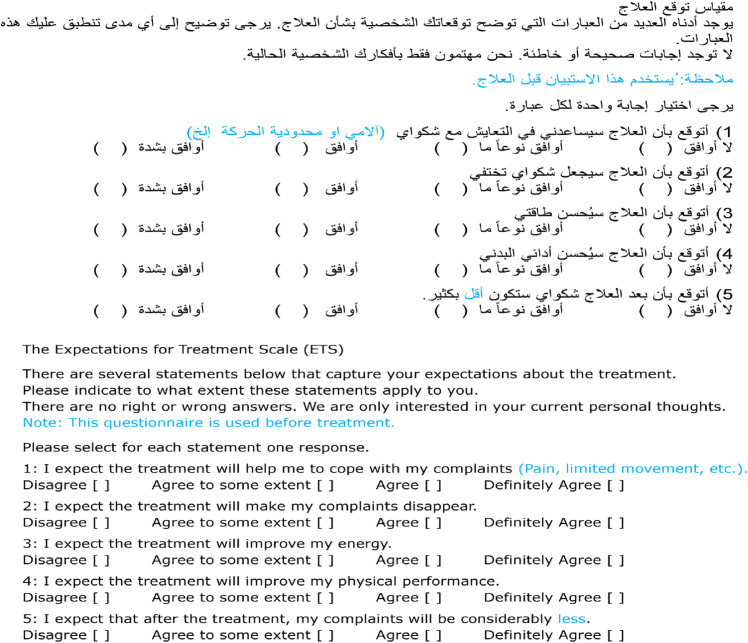
English and Arabic versions of the final translated culturally adapted ETS. Note: text highlighted in blue is where amendments were made during the cultural adaptation process.

During the adaptation phase, the Arabic ETS underwent specific modifications: a) replacing the word أفضل (better) with أقل (less) in item 5, b) removing the letter Haa (ه) from بأنه (that) in item 1, and c) incorporating a note in the instructions to clarify that this questionnaire is used before treatment. S3 Appendix offers a comprehensive overview of the proposed amendments, the adaptation committee decision, a description of the decision, and a comparison of the original phrases from the questionnaire with the updated versions following the approved modifications. Fig 5 provides Arabic and English versions of the final translated culturally adapted ETS.

### Validation results

A total of 205 individuals completed the questionnaire online with a mean age of 44.2 (18–86, SD = 16.45), and 52.2% (107 males and 47.3% (98) females (Table 2). Only 36 patients (17.5%) completed the questionnaire for the second time for test-retest reliability. Table 3 demonstrates the number of participants and statistical methods used to evaluate different psychometric properties.

**Table 2:** Characteristics of the participants in the validation phase.

Variable		Value
Age (mean ± SD), years		44.22 ± 16.45
Age range (min–max)		18–86
Male, n (%)		107 (52.2%)
Female, n (%)		98 (47.8%)
Education level, n (%)	Bachelor or Diploma	76 (37.1%)
	Master’s degree	41 (20.0%)
	Prefer not to say	33 (16.1%)
	High School	20 (9.8%)
	Primary School	15 (7.3%)
	Secondary School	11 (5.4%)
	PhD	9 (4.4%)

**Table 3:** Number of participants and statistical methods used for psychometric properties evaluation.

Psychometric Property	Statistical Test/ Index	n
Internal Consistency	Cronbach’s α	205 (patients)
Test–Retest Reliability (Total Score)	ICC (2-way random, absolute agreement)	36 (patients)
Test–Retest Reliability (Item level)	Weighted Kappa (κw)	36 (patients)
Measurement Error	SEM	205 (patients)
	SDC	205 (patients)
Content Validity	I-CVI and S-CVI	10 (physiotherapists)
Construct Validity (Known-Groups)	Pearson’s r	205 (patients)
Structural Validity	Exploratory Factor Analysis	205 (patients)
Cross-Cultural Validity	Differential Item Functioning	205 (patients)
Floor Effect	% minimum score	205 (patients)
Ceiling Effect	% maximum score	205 (patients)

Cronbach’s α = Cronbach’s Alpha; ICC = Intraclass Correlation Coefficient; SEM = Standard; Error of Measurement; SDC = Smallest Detectable Change; I-CVI = Item-Level Content Validity Index; S-CVI = Scale-Level Content Validity Index.

#### Face validity.

All interviewees, apart from 7, stated that the Arabic ETS is thorough and covers their expectations concerning the outcomes of physiotherapy. Participant 7 acknowledged a flaw in the questionnaire but could not identify or provide any additional concepts for inclusion.

Participant 13: “*Yes, in my opinion, the questionnaire is comprehensive and covers patient expectations about the treatment outcomes*.”

Participant 19: “*Of course, my expectations were the same as what was stated in the questionnaire.*”

#### Content validity.

The I-CVI values ranged from 0.6 to 0.9 (Table 4), with the overall S-CVI (calculated as the mean of the I-CVI values) of 0.78. Which is considered acceptable, considering that 10 physiotherapists evaluated the content validity [42].

**Table 4:** Expert ratings, item-level (I-CVI) and scale-level (S-CVI) content validity indices for the arabic ETS.

Item/ Expert	Expert 1	Expert 2	Expert 3	Expert 4	Expert 5	Expert 6	Expert 7	Expert 8	Expert 9	Expert 10	Agreement	I-CVI
Item 1	1	1	1	1	0	0	1	1	1	1	8	0.8
Item 2	1	1	1	1	1	0	1	1	1	1	9	0.9
Item 3	0	0	1	1	1	0	0	1	1	1	6	0.6
Item 4	1	0	1	1	0	0	1	1	1	1	7	0.7
Item 5	1	1	1	1	1	0	1	1	1	1	9	0.9
Arabic ETS Content Validity Index (S-CVI)												0.78

#### Construct validity.

For known-groups validity, a bivariate correlation analysis demonstrated a statistically weak (r = −0.331, p < .001) negative correlation between age and level of expectations [46], with the overall expectations score decreasing as age increases, which aligns with the initial hypothesis. For structural validity, Kaiser-Meyer-Olkin measure of 0.78 implies an appropriate sample size for factor analysis [47,48], with a Bartlett’s Test of Sphericity of 218.73 (p < 0.001), implying a strong correlation among the items and making them suitable for factor analysis [47]. Only item 1 had an eigenvalue above 1 (2.53), which suggests the unidimensionality of the ETS (Kaiser, 1970). DIF analysis partially supported the cross-cultural validity, demonstrating partial measurement invariance among males and females. In the Chi-Square test in crosstabs for DIF, with three degrees of freedom, the p-values for items 1, 3, and 5 are all greater than 0.05 (0.76–0.27, and 0.10, respectively). However, two items (Items 2 and 4) showed significant DIF, with values of 0.008 and 0.027, respectively.

#### Reliability.

The ETS Arabic measure demonstrated good internal consistency, with a Cronbach’s Alpha of 0.75. The “Cronbach’s Alpha if Item Deleted” values for items 1–5 were 0.71, 0.71, 0.69, and 0.72, respectively. Pearson correlation coefficient ratios varied between r = .292 to r = .478. With an average of 13 days interval, results show a high level of test-retest reliability with an ICC of 0.93 (95% CI: 0.86–0.96, p < .001), and a Weighted Kappa items 1–5 of 0.75, 0.58, 0.55, 0.66, 0.70, respectively (Table 5). The SEM for the Arabic ETS scale was low (1.42), and the SDC was 2.86. The Limits of Agreement (LOA) values were 14.89 (SD = 2.86). The 95% LOA were computed as mean difference ± 1.96 × SD = 14.89 ± 5.61, resulting in a range of 9.27 to 20.51.

**Table 5:** Cohen’s Weighted Kappa for retest reliability for Items 1 to 5 (average of 13-day interval)*.*

Items (test-retest)	κw^a^	Std. Error^b^	z^c^	P-value
**Item 1**	.75	.09	6.54	<.001
**Item 2**	.58	.11	4.77	<.001
**Item 3**	.55	.11	4.45	<.001
**Item 4**	.66	.11	5.46	<.001
**Item 5**	.70	.10	6.24	<.001

^a^The estimation of the weighted kappa uses linear weights.

^b^Value does not depend on either null or alternative hypotheses.

^c^Estimates the asymptotic standard error assuming the null hypothesis that weighted kappa is zero.

#### Floor and ceiling effects.

**Fig 6:**
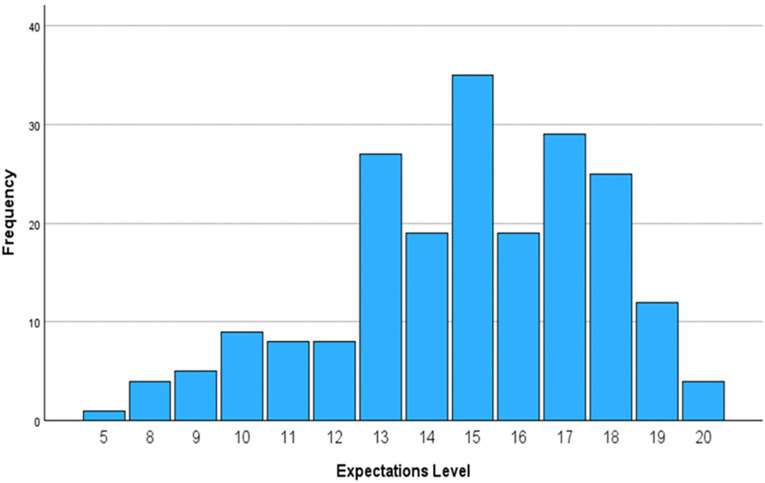
Distribution of total expectations scores of the Arabic ETS.

There were only very small floor and ceiling effects that could be observed. More precisely, only 0.5% of the participants achieved the lowest score of 5, which indicates a small floor effect. This suggests that the measure is successful in accurately capturing the lower levels of outcome expectations. In addition, a small ceiling effect was seen, with 2% of respondents scoring at the highest possible value. This shows adequate sensitivity in differentiating participants at the top range of the scale (Fig 6).

## Discussion

This study presents a culturally adapted and validated Arabic version of the ETS. This study utilised a systematic translation process that included bilingual translators, with two translators assigned for both forward and backward translations. Each translation phase was then evaluated by a synthesis. Interviewees’ perspectives and suggested changes were also addressed during cross-cultural adaptation, which was followed by an extensive psychometric evaluation. The results demonstrated that the Arabic ETS displayed a significant degree of semantic and conceptual equivalence with the original English version and provided substantial evidence for its psychometric properties.

The Arabic ETS demonstrated good face validity, acceptable content validity, confirmed (though not definitive) known-group validity (results aligning with the hypothesis), confirmed structural validity (EFA identified a unidimensional structure), partially supported cross-cultural validity, good internal consistency and high test-retest reliability. The measurement error for an individual’s score on the Arabic ETS scale was small, approximately 1.42 points on average, with a SDC of 2.86 points (95% CI). The ETS was not translated into other languages so the psychometric properties of the Arabic version can be compared to those of other languages versions.

There are many published guidelines for translating and culturally adapting self-reported measures. This study follows the guidelines developed by Tsang et al. [27], which are frequently referenced for the translation and cultural adaptation of surveys, especially in health and social sciences. These criteria encompass systematic methodologies aimed at maintaining the content validity, reliability, and relevance of surveys across many languages and cultural contexts [27,32]. However, Epstein et al. [49] conducted a critical evaluation of the guidelines for the translation and adaptation of self-reported measures, highlighting that while the Beaton model is widely employed, it has several limitations. A significant issue is the complexity and resource demands of the five-stage process, which may be impractical for researchers with little funding [49]. Epstein et al. [49] assert that research has yet to reach a consensus on the most effective translation method, as each guideline has unique advantages and limitations.

In the scope of the translation, cross-cultural adaptation and validation of self-reported measures, the findings of our study align with various key concerns highlighted in the relevant literature. Although it was not significant, translating the ETS into Arabic involved difficulties similar to those described by previous research [32,50]. For example, maintaining conceptual equivalence of the ETS presented a challenge, an obstacle that is extensively documented in cross-cultural adaptation studies. The adaptation committee changed the wording of Item 5 (I expect that after the treatment, my complaints will be considerably better) and replaced ‘’better” with ‘’lesser.” Five interviewees suggested that the word ‘’better” in Arabic may be understood as more, so they asked for this change. Therefore, if left as it is, item 5 may be understood as the complete opposite, and participants may understand it as asking that they ‘’will have more complaints.” The difficulties of keeping the conceptual meaning and translating culture-specific concepts into Arabic are also highlighted in previous research [51,52]. For example, in a study translating the Post-Study System Usability Questionnaire into Arabic, the word “pleasant” in one of the items was translated into “interesting” and “enjoyable” by different translators. However, the committee refused both options and proposed the word “satisfying” instead [52].

The two backward translators did not know the questionnaire’s intended concept to prevent bias (Beaton et al., 2000). Although it is not clearly stated what type of bias this may be, it could be what is known as “confirmation bias”. Among a few types of human cognitive biases, the literature identifies “confirmation bias” as the strongest and most prevalent [53]. Confirmation bias is when individuals look for confirmation of their beliefs instead of invalidating and contradicting their preexisting presumptions [53]. Confirmation bias implies the inconsistent leaning towards and prioritising evidence that supports one’s established preconceived beliefs while minimising and overlooking the contradictory evidence [54]. Minimising confirmatory bias by blinding back translators to the measure’s conceptual bases may have enhanced the validity of the translation process and therefore the linguistic accuracy of the Arabic ETS. However, the fundamental assessment of a translated measure resides in its psychometric performance; examining its psychometric properties will ensure its suitability and reliable usage for the target audience.

The Arabic ETS exhibited robust psychometric properties. Cronbach’s alpha was 0.75, indicating satisfactory internal consistency. Additionally, ‘’Cronbach’s Alpha if item deleted” values were all found to be lower than the overall Cronbach’s alpha, meaning that each item positively contributed to scale reliability. This suggests that the 5 items collectively measure the same underlying construct (outcome expectations). However, Cronbach’s alpha, although commonly utilised to assess the internal consistency of scale items, has limitations [55]. Cronbach’s alpha value may be falsely high if the items exhibit significant semantic or conceptual overlap [55]. In other words, a high Cronbach’s alpha may not necessarily indicate that your scale is genuinely reliable; it could simply suggest that the items are redundant. Therefore, the inter-item correlation between items was evaluated using Pearson’s correlation. The highest correlation (r = 0.47) was between items 2 and 3, suggesting no significant redundancy [56].

The item-level reliability was moderate to strong, as evidenced by weighted kappa values. Reliability was confirmed by the fact that all coefficients were statistically significant (p < .001). The results indicate that the average measurement error for an individual’s score on the Arabic ETS scale is around 1.42 points and for a change in an individual’s score to be deemed a “real” change beyond the measurement error, it must exceed 2.86 points (CI 95%). The score variability was acceptable, although somewhat broad, as evidenced by LOA values.

Content validity was confirmed by S-CVI of 0.78 and I-CVI of 0.6 to 0.9, which indicates acceptable content validity [42]. This suggests that the 5 items are relevant and complete for outcome expectations. Using a consistent content validity form ensured that raters (physiotherapists) understood their roles, reducing bias in item evaluation. Nevertheless, the generalisability of the content validity of the Arabic ETS was restricted by the fact that only physiotherapists evaluated content validity. However, item 3 seems to have a lower rating compared to the other 4 items in the Arabic ETS. This may be attributed to a variety of reasons, including the wording of the item. As suggested by one of the interviewees, the word طاقتي (“my energy”) may be replaced with قدرتي (“my capability”) for better understanding. However, this suggested change was not implemented as the adaptation committee argued that capability and energy are two distinct constructs, and it was only suggested by one out of 24 interviewees.

Construct validity was verified through hypothesis testing for known-group validity, EFA for structural validity, and DIF for cross-cultural validity. Supporting known-group validity of the Arabic ETS, findings were consistent with the hypothesis that younger individuals scored higher [43], which may be attributed to greater exposure to health-related information and more proactive healthcare attitudes [12]. The EFA confirmed unidimensionality of the structure of the ETS. Although unidimensionality simplifies scoring and interpretation [57], it may obscure significant nuances in patient expectations that may be pertinent in particular contexts or subpopulations. To verify this structure in independent samples and investigate whether multidimensional models provide supplementary insights, confirmatory factor analysis (CFA) may be undertaken in future research. CFA is more effective at testing unidimensionality than EFA [57]. However, considering that expectations may be influenced by factors such as education level, age and the severity of the condition [43,58], selecting age as the sole grouping variable for evaluating known-group validity may be another limitation of this research.

Cross-cultural validity was partially supported by the findings. Cross-cultural validity of Items 1, 3, and 5 was supported by the absence of significant sex (male, female, prefer not to say) differences (p > 0.05) in the DIF analysis. Conversely, Items 2 and 4 demonstrated substantial DIF (p = 0.008 and 0.027), suggesting potential cultural interpretation discrepancies. However, the Arabic ETS’s cross-cultural validity can be further established by the utilisation of a multi-step procedure that included independent forward and backward translations, expert committee evaluation, and pilot testing [59], following internationally recognised cross-cultural adaptation guidelines [27]. These strategies ensured the conceptual equivalence and cultural relevance of the Arabic ETS.

Lastly, comparable to the original version [21], the Arabic ETS exhibited minimal floor and ceiling effects. Ceiling effects are a common issue when evaluating patient expectations, as individuals pursuing an intervention generally anticipate substantial outcomes; otherwise, they would not have the incentive to seek the intervention [21]. Some outcome expectations measures, such as the Acupuncture Expectancy Scale, demonstrate significant ceiling effects (36–49%), whilst others, such as the HSS ACL Reconstruction Preoperative Expectations Survey [60] and the HSS Knee Replacement Expectations Survey [61], displayed 15% of the ceiling effect. The EXPECT Questionnaire [62] and the HSS Cervical Spine Surgery Expectations Survey [63] have minor ceiling effects (3% and 4%, respectively), similar to the Arabic ETS, indicating superior sensitivity in assessing subtle differences and the full spectrum of patient expectations.

### Limitations

There are some limitations of this research. Referring to the guidelines, which advise a sample size of 30–50 [27], the sample size for the adaptation phase may be small (24). Since the adaptation process is heavily reliant on participant feedback [27,32], the quality, reliability, and validity of the adapted instrument are restricted by the limited number of patients recruited. Hall et al. [64] indicate that there is no consensus on the ideal sample size for piloting and adjusting a questionnaire, which often varies from 5 to 50 participants.

There was a significant dropout rate in the number of participants filling the questionnaire for the second round (test-retest). Only 36 participants out of 205, which results in a dropout rate of approximately 82.4%. Dropout in longitudinal research or research that demands follow-ups may include research design burden, loss of motivation, lack of interest in the research/intervention, time restrictions, and health and life consequences [65–67].

Within the psychometric evaluation literature, determining the sample size is a significant challenge for researchers and psychometricians [68]. When evaluating test-retest reliability, the necessary sample sizes are relatively small [69]. The required sample sizes for test-retest reliability assessed by the kappa agreement and intra-class correlation are 15 and 22 participants, respectively [69]. Incorporating a non-response rate of 20.0%, a minimum sample size of 19 is needed for Kappa agreement and 28 is needed for ICC [69]. Meaning that a sample size of 36 for the test-retest reliability is arguably sufficient and may not have affected the rigour of the findings.

Although the main purpose of this study was to alter the questionnaire for Arabic speakers, it is vital to note that the recruitment was limited to individuals from Libya. The Arabic language contains several dialects, demonstrating regional variations across numerous nations and localities [70]. However, the language used to translate the questionnaire was the modern standard Arabic. Arabic is often categorised into three principal variants: (i) Quranic or Classical Arabic; (ii) Modern Standard Arabic, utilised throughout many media forms such as news, films, translations, and literature; and (iii) Colloquial or Daily Arabic [71]. Modern Standard Arabic is considered the most widely spoken dialect in several Arab countries [71,72]. Moreover, one forward and one backward translator were from Saudi Arabia, which may help in ensuring that the translated ETS may also be suitable for other Arabic-speaking communities.

A procedural limitation of this research would be that backward translators were involved in the forward translation synthesis. While not blinding backward translators to the forward translation versions may be considered a deviation from some of the guidelines [33], this does not necessarily compromise the reliability of the translation process. However, we undertook a comprehensive committee evaluation following the back-translations that would arguably have mitigated any possible influence, arising from the lack of blinding [49].

Some guidelines recommend that back translators be blinded to any version of the measure [33], while others recommend blinding them only to the original text (original version before forward translation) [32] and not necessarily from the forward translations. In this research, back transistors were blinded to the original ETS. A systematic review of the guidelines for the translation and cross-cultural adaptation of self-reported measures [49] argues that the translation process was improved by the expert committee, rather than back-translation. Therefore, in this research, the translation committee met following each translation.

Moreover, the results demonstrate partial support for the cross-cultural validity of the Arabic ETS. The DIF analysis indicated no significance for three items (1, 3, and 5, p > 0.05), suggesting these items function similarly across genders (males and females). However, notable DIF was identified for items 2 and 4, indicating possible differences between groups’ (males and females) interpretation or responses to these two items. This unequal functioning of these two items may indicate social gender norms in Arabic-speaking populations that influence the interpretation of outcome expectations, or minor linguistic nuances in the translated ETS, which are interpreted differently by males and females. These two items may also reflect aspects of outcome expectations that manifest somewhat differently across genders, rather than reflecting mere item bias. This underlines the necessity for additional assessment or possible refinement of items 2 and 4 of the Arabic ETS to guarantee comprehensive cross-cultural validity. While the current findings support the use of the Arabic ETS, it may require further research across a variety of clinical conditions to further examine its measurement invariance.

Lastly, there were some challenges in evaluating some psychometric properties. Evaluating convergent validity for the Arabic ETS was not possible because there is no other Arabic outcome expectations measure. Similarly, the responsiveness of the Arabic ETS was not assessed. Responsiveness should be evaluated in a longitudinal approach [73], in which hypotheses are examined like in construct validity [74]. The construct validity in this research has been assessed through three methods; consequently, evaluating responsiveness (longitudinal construct validity) may be unnecessary.

## Conclusion

The Expectation for Treatment Scale (ETS) was successfully translated and adapted to the Arabic culture. The ETS was modified to ensure that it is consistent with the Arabic cultural context while maintaining its original purpose. The effective translation and cultural adaptation of the ETS allow its relevance and application within the target population. The findings provide substantial evidence for the psychometric properties of the Arabic ETS that ensure its reliable and valid use to measure patients’ outcome expectations in Arabic-speaking patients with MSDs. The validation of the Arabic ETS opens the avenues for its broader use in clinical and research settings, considering that it is a generic measure and the first valid measure to measure outcome expectations for Arabic speakers. This may also allow future research to investigate cultural variations in outcome expectations between Arabic and other cultures.

## Supporting information

S1 Appendix Interview questions. (DOCX)

S2 Appendix Translated (Arabic) ETS before adaptation. (DOCX)

S3 Appendix A comprehensive overview of the interviewees’ proposed amendments, a committee decision, a description of the decision, and a comparison of the original phrases from the questionnaire. (DOCX)
