# Supplementary material for: Arabic translation, cross-cultural adaptation, and validation of the Expectation for Treatment Scale (ETS) in patients with musculoskeletal disorders
Source: PLoS One. 2026 Mar 27;21(3):e0346025. doi: 10.1371/journal.pone.0346025 (PMC13028328; doi:10.1371/journal.pone.0346025)
Supplement: S1 Appendix — (DOCX) [file pone.0346025.s001.docx]

Appendix 1: Interview questions.

| Question | | | Additional prompts |
| --- | --- | --- | --- |
| Adaptation questions | Q1 | What are your thoughts about the questionnaire instructions? | - Did you have any difficulties in following the questionnaire instructions? |
|  | Q2 | What are your thoughts about the questionnaire language? | - Is the questionnaire language understandable?  - Are there any difficult words or phrases? If yes, please explain |
|  | Q3 | Would you like to rephrase any of the questions in your own words because the wording is not well understood? | - If yes, in what way? Please, can you elaborate? |
|  | Q4 | Would you change any of the words to make the questions more understandable and acceptable? | - If yes, please explain |
|  | Q5 | What do you think about the response options? | - Were the question responses simple to use?  - Was it simple to respond to the questions? |
|  | Q6 | Do you have any remarks regarding the response options? | - If yes, please explain |
|  | Q7 | Is there anything else you would like to add about the questionnaire? |  |
| Face validity questions | Q8 | To what extent do you think the questions cover your expectations of treatment outcomes? | - Can you please grade its comprehensibility on a scale of 1-4? |
