# Supplementary material for: Arabic translation, cross-cultural adaptation, and validation of the Expectation for Treatment Scale (ETS) in patients with musculoskeletal disorders
Source: PLoS One. 2026 Mar 27;21(3):e0346025. doi: 10.1371/journal.pone.0346025 (PMC13028328; doi:10.1371/journal.pone.0346025)
Supplement: S2 Appendix — (DOCX) [file pone.0346025.s002.docx]

Appendix 2: Translated (Arabic) ETS before adaptation.

**مقياس توقع العلاج**

**يوجد أدناه العديد من العبارات التي توضح توقعاتك الشخصية بشأن العلاج. يرجى توضيح إلى أي مدى تنطبق عليك هذه العبارات. لا توجد إجابات صحيحة أو خاطئة. نحن مهتمون فقط بأفكارك الشخصية الحالية.**

**يرجى اختيار إجابة واحدة لكل عبارة.**

1. أتوقع بأن العلاج سيساعدني في التعايش مع شكواي

لا أوافق ( ) أوافق نوعاً ما ( ) أوافق ( ) أوافق بشدة ( ) 

1. أتوقع بأن العلاج سيجعل شكواي تختفي

لا أوافق ( ) أوافق نوعاً ما ( ) أوافق ( ) أوافق بشدة ( )

1. أتوقع بأن العلاج سيُحسن طاقتي

لا أوافق ( ) أوافق نوعاً ما ( ) أوافق ( ) أوافق بشدة ( )

1. أتوقع بأن العلاج سيُحسن أدائي البدني

لا أوافق ( ) أوافق نوعاً ما ( ) أوافق ( ) أوافق بشدة ( )

1. أتوقع بأنه بعد العلاج شكواي ستكون أفضل بكثير

لا أوافق ( ) أوافق نوعاً ما ( ) أوافق ( ) أوافق بشدة ( )

**English version of the Translated (Arabic) ETS before adaptation.**

**There are several statements below that capture your expectations about the treatment.** **Please indicate to what extent these statements apply to you.** **There are no right or wrong answers. We are only interested in your current personal thoughts.**

**Please select for each statement one response.**

1: I expect the treatment will help me to cope with my complaints.

Disagree [ ] Agree to some extent [ ] Agree [ ] Definitely Agree [ ]

2: I expect the treatment will make my complaints disappear.

Disagree [ ] Agree to some extent [ ] Agree [ ] Definitely Agree [ ]

3: I expect the treatment will improve my energy.

Disagree [ ] Agree to some extent [ ] Agree [ ] Definitely Agree [ ]

4: I expect the treatment will improve my physical performance.

Disagree [ ] Agree to some extent [ ] Agree [ ] Definitely Agree [ ]

5: I expect that after the treatment, my complaints will be considerably better.

Disagree [ ] Agree to some extent [ ] Agree [ ] Definitely Agree [ ]
