# Supplementary material for: Arabic translation, cross-cultural adaptation, and validation of the Expectation for Treatment Scale (ETS) in patients with musculoskeletal disorders
Source: PLoS One. 2026 Mar 27;21(3):e0346025. doi: 10.1371/journal.pone.0346025 (PMC13028328; doi:10.1371/journal.pone.0346025)
Supplement: S3 Appendix — (DOCX) [file pone.0346025.s003.docx]

Appendix 3: A comprehensive overview of the interviewees' proposed amendments, a committee decision, a description of the decision, and a comparison of the original phrases from the questionnaire.

| Suggested change | | Participants | Place of suggested change | Committee decision | Description | Before changing | After changing | Remarks |
| --- | --- | --- | --- | --- | --- | --- | --- | --- |
| 1 | Removing the letter Haa (ه) from بأنه (that). | 1,7 | Item 5 | Agree | The only difference in her is that they recommended removing the connected pronoun Haa (ه), which is arranged in the accusative case (نصب) with respect to the noun of the noun that follows it. | 5) I expect that after the treatment, my complaints will be considerably better. | 5) I expect that after the treatment, my complaints will be considerably less. | They proposed this to make the statement easier to understand, but it doesn't affect its meaning. |
| 2 | Replacing شكواي (my complaint) with آلامي (my pain). | 4,8 | All items | Don’t agree | The committee was opposed to this modification on the grounds that complaints are not limited to pain, unless the intention was to restrict the use of this questionnaire to individuals experiencing pain. The committee reached a consensus on including a clarification term to inform individuals that their complaint could be due to discomfort or a limitation. |  |  |  |
| 3 | Replacing التعايش (cope) with التأقلم (adapt). | 4 | Item 1 | Don’t agree | The committee was opposed to this modification on the grounds that "cope" would have a more negative connotation than "adapt," and that substituting the word would not alter its meaning in Arabic. |  |  |  |
| 4 | Replacing the word أفضل (Better) with أقل (Less). | 6,8,10, 16,24 | Item 5 | Agree | The committee collectively agreed to this modification because, if left unchanged, some patients might interpret the item as having the exact opposite meaning in Arabic. | 5) I expect that after the treatment, my complaints will be considerably better. | 5) I expect that after the treatment, my complaints will be considerably less | Five (20.8%) participants thought the phrase implied they expected greater complaints following treatment. One of the backword translators also raised this at synthesis. |
| 5 | Adding questions: | | | | | | | |
|  | A question that asks if the participant expects the treatment to worsen their complaints or situation. | 7 |  | Don’t agree | The committee disagreed with this addition, arguing that a treatment that exacerbates the patient's condition is not aligned with healthcare objectives and intentions. Furthermore, the developer of the original questionnaire argues that if a patient anticipates that the treatment will worsen their condition, they are unlikely to seek this particular treatment. |  |  |  |
|  | Three participants have proposed the inclusion of a question in the questionnaire acknowledging its brevity and its limitations. However, they were unable to propose any ideas for additional goods. | 7,8,15 |  | Don’t agree | The committee has decided not to pursue this comment because participants suggesting this lack a specific suggestion regarding which part of their outcomes expectations is missed in the questionnaire and may therefore be included in the questionnaire. |  |  |  |
|  | A question about duration of treatment, along with proposed timelines for observing progress, so, individuals have the option to select a certain timeframe according to their expected improvement. | 15 |  | Don’t agree | The committee disapproved this addition, arguing that the treatment length is not a relevant to the aspect of treatment outcome expectations. |  |  |  |
|  | A question asking about Clinic’s equipment and readiness. | 6 |  | Don’t agree | The committee disapproved this addition, arguing that readiness of the clinic and the clinicians are not a relevant the aspect of treatment outcome expectations. |  |  |  |
| 6 | Not easy to read and understand. | 8 | Item 5 | Don’t agree | The committee rejected this addition, citing that none of the other 23 participants encountered any challenges in comprehending this item. Furthermore, this item has undergone two modifications in this process, which would likely enhance its suitability and readability. |  |  |  |
| 7 | Adding ‘I don’t know’ response option. | 8 | All items | Don’t agree | The expert committee disagreed with this addition, expressing that the same participant indicated in a subsequent question that it was not challenging to answer the question or select a response option. Furthermore, they reported founding the response option that accurately represented their expectations or what they intended to convey about their expectations.  The committee discussed that when considering expected treatment outcomes, absolute confidence about future outcomes is unattainable. This remark acknowledges the inherent constraints of human comprehension. Despite the uncertainty, it is crucial to make decisions based on the most precise and thorough information available. This process allows us to make decisions carefully and thoughtfully, even when the end outcome is uncertain. |  |  |  |
| 8 | Adding a statement to clarify that this questionnaire is used before treatment. | 8,11 | Start of the questionnaire | Agree | The expert committee unanimously approved this modification, arguing that it has no impact on the actual questionnaire and will enhance people's comprehension of its intention. |  | Note: This questionnaire is used before treatment. | Note: this questionnaire is used before treatment. |
| 9 | Replacing طاقتي (my energy) with قدرتي (my capability). | 10 | Item 3 | Don’t agree | The committee was opposed to this modification on the grounds that substituting the word would not alter its meaning in Arabic.  The committee argued that the English language contains a distinction between capability and energy constructs. Moreover, capability and energy are distinct concepts in Arabic, and this is an aspect of the language that translates smoothly. While the concept of energy incorporates both physical and psychological aspects, the term "energy" would most probably be interpreted as referring to physical energy, which signifies the ability to execute a given task and thus assigns a degree of reference to capability. Therefore, the committee concluded that the proposed change lacks merit. |  |  |  |
| 10 | Similar questions | 12,19 | Items 1, 2 and 5 | Don’t agree | The expert committee has deliberated on these two items, contending that while they bear resemblance, they are not identical. The committee has reached a consensus that there is a distinction between  سيجعل شكواي تختفي (To make my complaints disappear) and  شكواي ستكون أقل بكثير (to make my complaint considerably better). The difference resides in the entire elimination of the complaint compared to a substantial reduction, particularly when dealing with chronic conditions.  The committee recognised that item 1 expresses a distinct expectation in comparison to items 2 and 5, namely that the treatment will aid in the management of the individual's complaints. The committee deliberated that item 1 implies a less ambitious expectation. It suggests that the treatment may not necessarily eliminate or substantially reduce the complaint, but rather make it easier to manage or less burdensome. |  |  |  |
| 11 | replacingأوافق نوعا ما )partially agree( with  ربما أوافق )I maybe agree). | 19 | All items | Don’t agree | The expert committee has deliberated on these two alternatives and contended that the term ربما )maybe( possesses a certain degree of probability. It is comparable to the intensity of one's expectation or the degree to which one expects to convey precisely what is being expressed. |  |  |  |
